# The clinical features of familial focal epilepsy with variable foci and *NPRL3* gene variant

**DOI:** 10.1371/journal.pone.0284924

**Published:** 2023-04-26

**Authors:** Yue Wang, Peimin Yu, Guoxing Zhu, Xunyi Wu, Ding Ding, Zhen Hong

**Affiliations:** Department of Neurology, Huashan Hospital, Fudan University, Shanghai, China; Policlinico Riuniti of Foggia: Neuroscience Department, S.C. Ospedaliera of Neurology-Stroke Unit, ITALY

## Abstract

**Objective:**

Familial focal epilepsy with variable foci (FFEVF) is a rare type of focal epilepsy syndrome; it is associated with *NPRL3* variant. However, relevant reports are rare in China. We aimed to analyze the clinical features of Chinese patients with FFEVF to understand further the differences between various *NPRL3* variants and explored the effect of *NPRL3* variant on mRNA.

**Methods:**

We ran a full workup on a family with FFEVF (four patients, one healthy member): an inquiry of medical history, cranial magnetic resonance imaging (MRI), electroencephalogram (EEG), and whole exon sequencing. Their clinical features were compared with those of other FFEVF patients in published reports. The mRNA splicing changes were analyzed quantitatively and qualitatively using real-time quantitative—polymerase chain reaction (q-PCR) and reverse transcription (RT)-PCR and compared between our patients and healthy individuals.

**Results:**

Patients with *NPRL3*: c.1137dupT variant had a wide range of onset age (4 months to 31 years), diverse seizure types, variable foci (frontal lobe/temporal lobe), different seizure times (day/night) and frequencies (monthly/seldom/every day), different therapeutic effects (refractory epilepsy/almost seizure free), normal MRI, and abnormal EEG (epileptiform discharge, slow wave). The phenotypic spectrum with different *NPRL3* variants was either similar or different. Significantly different relative quantities of mRNA were found between patients and healthy individuals in real-time qPCR. Abnormal splicing was observed in patients compared with healthy individual in RT-PCR. Despite having the same gene variant, different family members had different mRNA splicing, possibly causing different phenotypes.

**Conclusion:**

The clinical features of FFEVF varied, and auxiliary inspection was atypical. *NPRL3*: c.1137dupT could change the relative quantity of mRNA and cause abnormal splicing, which might produce different phenotypes in different family members.

## Introduction

Epilepsy is a neurological condition that is defined as the presence of any of the following: (1) At least two unprovoked (or reflex) seizures occurring >24 h apart. (2) One unprovoked (or reflex) seizure and a probability of further seizures similar to the general recurrence risk (at least 60%) after two unprovoked seizures, occurring over the next 10 years. (3) Diagnosis of an epilepsy syndrome [1]. The etiologies can be structural, genetic, infectious, metabolic, immune, or unknown, according to the International League Against Epilepsy (ILAE) classification, 2017 [2–4].

Among them, genetic causes have the greatest influence in determining the onset of epilepsy. Familial focal epilepsy with variable foci (FFEVF) is a rare autosomal dominant form of epilepsy characterized by complex and variable clinical manifestations and different epilepsy locations among family members, which can be identified based on the clinical features, neuroimaging and electroencephalogram (EEG).

The GTPase-activating protein activity toward Rags (GATOR) complex is located upstream of the mTORC1 signal transduction pathway and has amino acid receptor activity. It consists of two highly conserved protein complexes, GATOR1 and GATOR2, which act as negative and positive regulators of mTORC1 pathway activity, respectively. During embryonic brain development, the mTORC1 signaling pathway can respond to various extracellular stimuli, including growth factors, insulin, nutrients, amino acids and glucose. It plays a key regulatory role in cell proliferation, migration, axon and dendrite growth, and cortex formation [5]. Dishevelled, Egl-10 and Pleckstrin Domain Containing protein 5 (*DEPDC5)*. which encodes the GATOR1 complex, has been identified in more than 8% of FFEVF cases [6]. In recent years, the other two genes encoding the GATOR1 complex, nitrogen permease regulator-like 2 (*NPRL2*) and nitrogen permease regulator-like 3 (*NPRL3*), have also been found to be associated with FFEVF [7,8]. Most *NPRL3* variants produce nonfunctional proteins with abnormally short cycles, resulting in reduced normal GATOR1 complex formation, leading to overactivation of the mTORC1 pathway and signal transduction of the mTOR pathway.

However, few studies [9,10] on *NPRL3* have been conducted in China. This study aimed to describe a new *NPRL3* variant associated with FFEVF. We summarized the unique clinical characteristics of members of a family with FFEVF and expanded the clinical and molecular spectrum of *NPRL3*. Furthermore, our study explored the possible mechanisms underlying FFEVF caused by this variant, which might be important for understanding the disease and aid the clinical evaluation of anti-seizure medications, especially those targeting the defective mTOR pathway.

## Materials and methods

### Study population

The clinical data of the study group were collected from four patients from the same family and one unaffected relative who first visited the neurology clinic in Shanghai Huashan Hospital in April 2020. Seizures were classified according to the ILAE seizure classification. A total of three healthy participants were recruited to the control group in April 2020, all of whom were Han Chinese. They had no history of epileptic seizures within three generations and no neurological diseases, including febrile convulsions. The sex and age of all participants who underwent physical examination were matched with those of the patients in the study group. The examinees were not related to each other or to the members of the study group.

This study was conducted according to the principles of the Declaration of Helsinki and approved by the Ethics Committee of Shanghai Huashan Hospital (2020 clinical review No. 065). All the study participants signed informed consent forms. A copy of the consent form was provided to the participants via regular mail. During the interview, the participant confirmed that he/she had read and understood the consent form. If the participant had any questions about the information in the consent form, they were addressed. The participant stated that he/she was willing to participate under the conditions described in the consent form. The name of the participant, the consent date and time, and the participant’s oral consent were recorded.

### Participants’ characteristics

#### The clinical features of FFEVF and genetic testing

The epileptologists collected clinical information, including onset age, seizure type, course evolution, family history, and therapeutic effect of anti-seizure medications of all the patients in the family (I1, II3, II5, and III2) through face-to-face interviews. EEG and cranial MRI were performed. Peripheral blood samples of the family members (I1,II3, II4, II5, and III2) were collected for whole-exome sequencing (WES).

Illumina HiSeq X Ten systemI (llumina Inc, San Diego, CA, USA) was used to sequence genomic DNA libraries with acceptable concentrations and fragment sizes. The raw data quality was assessed and filtered to the reference genome. SNPs and InDels were identified with GATK 4 Haplotype Caller tool, and the variants were annotated using 1000 Genomes Project, Exome Aggregation Consortium, and Genome Aggregation Database. All screened variants were further confirmed using Sanger sequencing.

The pathogenicity of the gene variant was predicted via in silico analysis, whereas its clinical interpretation was based on the American College of Medical Genetics and Genomics criteria.

#### Differential RNA expression analysis of *NPRL3* variant

Blood samples (2–3 mL) of I1, II5, and III2 from the study group and the three healthy individuals in the control group were taken to extract RNA. According to the following reaction system (5*gDNA Eraser Buffer 2.0 μL, gDNA Eraser 1.0 μL, total RNA 350 ng, RNase free dH2O up to 10 μL), the reaction mixture was prepared on ice and reacted at 42°C for 2 min. Then, the reverse transcription reaction was formulated according to the following reaction system (the reaction liquid obtained in the previous step 10.0 μL, PrimeScript RT Enzyme Mix I 1.0 μL, RT Primer Mix 1.0 μL, 5* PrimeScript Buffer 2 (for real-time) 4.0 μL, RNase free dH2O 4.0 μL): 25°C for 5 min; 42°C for 30 min; 85°C for 5 min. The quantitative (Q)-polymerase chain reaction (PCR) was used to detect the cDNA expression levels (one pair of references and one pair of primers in each sample). The gene sequence of *NPRL3* was obtained from the NCBI GenBank database, and Primer 3.0 software was used to assist in the design of primers. The Q-PCR amplification system parameters were 2×SYBR Green Mix 5 μL, Primer-s 1 μL, Primer-as 1 μL, cDNA 2 μL, and ddH2O 1 μL. The concentration of Primer-s/Primer-as was 2.5 μM and the cDNA template was 10 times the diluent of the reverse transcribed stock solution. Q-PCR amplification was performed as follows: pre-denaturation at 95°C (3 min) and 40 cycles at 95°C (15 s) and 60°C (30 s).

#### Verification of *NPRL3* transcription level by splicing in vivo

Blood samples (2–3 mL) of I1, II5, and III2 were collected from the study group, and one blood sample of the same quantity was collected from the control group for RNA extraction as CK (Control Check). According to the reaction system (5*gDNA Erasor Buffer 2.0 μL, gDNA Erasor 1.0 μL, total RNA 7.0 μL, RNase free dH2O up to 10 μL), the reaction mixture was prepared on ice and reacted at 42°C for 2 min. The reverse transcription reaction was formulated according to the reaction system (the reaction liquid obtained in the previous step 10.0 μL, PrimeScript RT Enzyme Mix I 1.0 μL, RT Primer Mix 1.0 μL, 5* PrimeScript Buffer 2 [for real-time] 4.0 μL, RNase free dH2O 4.0 μL): 25°C for 5 min, 42°C for 30 min, and 85°C for 5 min. The obtained cDNA was used as the template for PCR amplification. Then, the amplified PCR products were sent to the gel sequencing for transcriptional analysis. Among them, the gene sequence of *NPRL3* was obtained from the NCBI GenBank database. Primer 3.0 software was used to assist in the design of primers. The PCR amplification system parameters were 2×Mix 15 μL, Primer-F 1 μL, Primer-R 1 μL, cDNA (wt/mut) 1 μL, and ddH2O 12 μL. PCR amplification was performed as follows: 95°C (5 min), 95°C (30 s), 57°C (30 s), and 72°C (1 min) for 30 cycles.

## Results

### The clinical features of FFEVF and genetic testing

**Fig 1:**
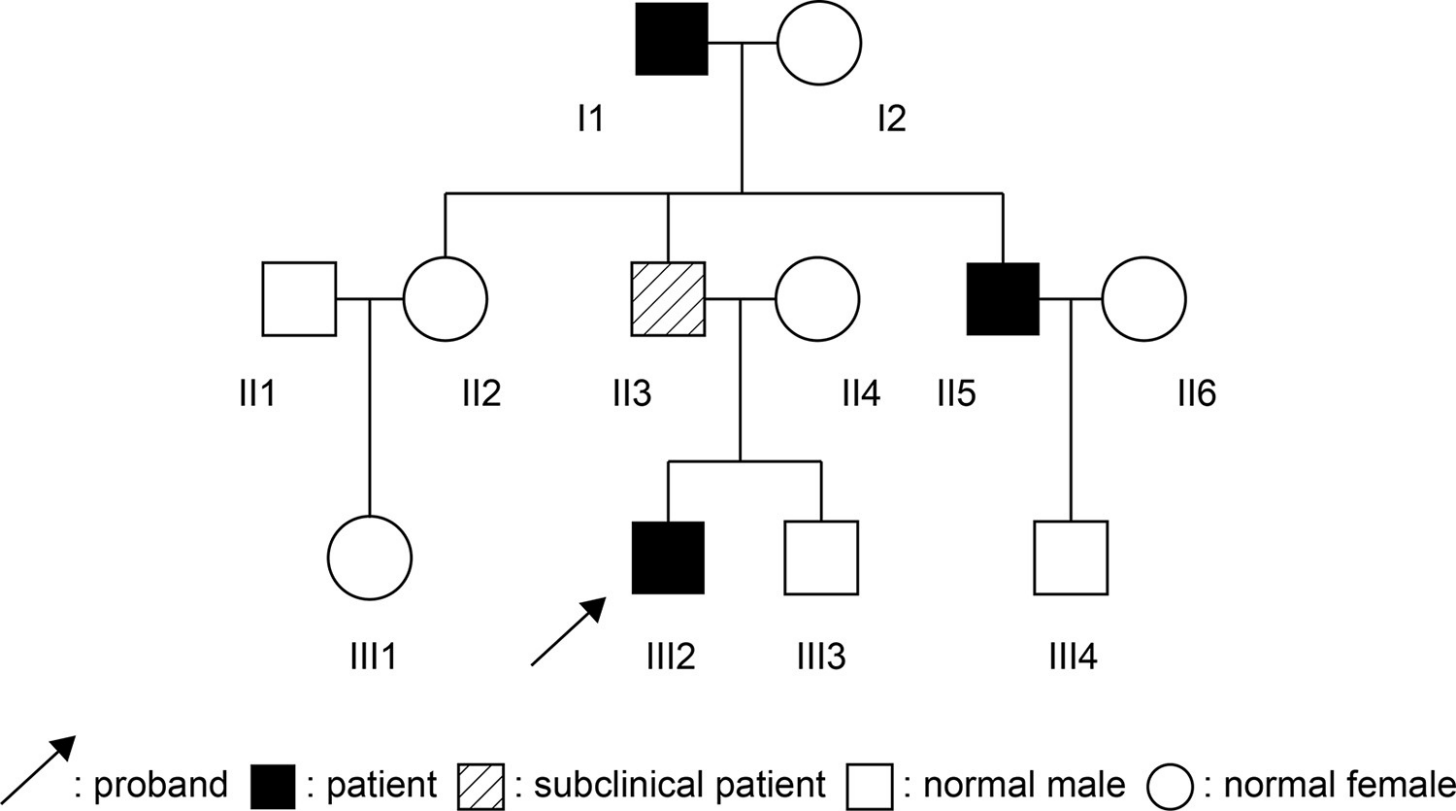
Pedigree. The family included several members presenting with focal epilepsy. Affected individuals are shaded and asymptomatic individuals are in white. Arrow indicates the proband.

There were four patients in this family, including three generations (Fig 1).

The onset age for the proband’s grandfather (I1) was 31 years. He suffered from nocturnal seizures characterized by complete impairment of consciousness and gaze deviation to the right, followed by bilateral tonic-clonic seizures that lasted 3–5 min. The seizure frequency was 1 to 10 times per month. He also had paroxysmal clouding of consciousness and automatism of the mouth and hands 2 to 3 times per month. MRI findings were normal, and EEG demonstrated bilateral sporadic and paroxysmal θ waves and sharp waves that were more apparent in the left temporal and frontal lobes. The prescribed medications were oxcarbazepine (OXC) 750 mg/d and sodium valproate sustained release tablets (VPA-SR) 2 g/d.

The proband’s father (II3) denied having any seizures. However, EEG demonstrated sporadic low-amplitude sharp waves in the bilateral temporal and occipital lobes.

The age at onset for the proband’s uncle (II5) was 6 years. He reported rare seizures characterized by an aura of a feeling of sadness, oral automatism, and breathlessness, followed by a bilateral tonic-clonic seizure, which lasted 3–5 min, in the daytime,. Occasionally, he experienced an aura (sad feeling). MRI findings were normal, and EEG demonstrated scattered low-amplitude sharp waves in the bilateral temporal lobes. The prescribed medications were OXC 1200 mg/d and VPA-SR 1 g/d.

The proband (III2) experienced paroxysmal blinking and loss of consciousness that was not related to fever at the age of 4 months. At 6 months, he experienced recurrent vomiting followed by a bilateral tonic-clonic seizure and loss of consciousness, unrelated to fever. From early childhood to the present, he reported experiencing loss of consciousness, eyes gazing to the right, and a bilateral tonic-clonic seizure, which was relieved 3–5 minutes later, every day. Most of the seizures were associated with sleep. MRI findings were normal and EEG demonstrated bilateral sporadic and paroxysmal θ waves, sharp waves, and sharp slow waves. All the lobes were involved. Despite receiving various anti-seizure medications (unspecified), he had seizures every day. He was taking OXC 1200 mg/d and VPA-SR 0.75 g/d at the time of the study, but the improvement was not apparent. He had a history of asphyxia at birth. His academic performance was poor, and he did not graduate from primary school.

The clinical features of the four patients available evaluated are included in Table 1.

**Table 1:** Clinical features of the patients in the study group.

Participant	Sex	Age at onset	Seizure type	Phenotype	Time of seizure	Seizure outcome	ASM use	MRI	EEG
I1	M	31 yr	FBTCS FS	FLE, TLE	Nocturnal	FBTCS: 1 to 10 times per month FS: 2 to 3 times per month	OXC, VPA-SR	N	Bilateral sporadic and paroxysmal θ waves, sharp waves, predominantly in the left temporal and frontal lobes
II3	M	--	--	--	--	--	--	N	Sporadic low-amplitude sharp waves, predominantly in the bilateral temporal and occipital lobes
II5	M	6 yr	FBTCS FS	TLE	Diurnal	Rare	OXC, VPA-SR	N	Low-amplitude sharp waves, predominantly in the bilateral temporal lobes
III2	M	4 mo	FS FBTCs	FLE, TLE	Nocturnal,diurnal	Every day	OXC, VPA-SR	N	Bilateral sporadic and paroxysmal θ waves, sharp waves, and sharp slow waves, involving all the lobes

Abbreviations: ASM: Anti-seizure medications; MRI: Magnetic resonance imaging; EEG: Electroencephalogram; M: Male; yr: Year; mo: Month; FBTCS: Focal to bilateral tonic-clonic seizure; FS: Focal seizure; FLE: Frontal lobe epilepsy; TLE: Temporal lobe epilepsy; OXC: Oxcarbazepine; VPA-SR: Sodium valproate sustained release tablets; N: Normal.

Through WES, an autosomal variant of *NPRL3*: c.1137 dupT was detected in the exon region of chromosome 16 (I1, II3, II5, III2). II4 had neither symptoms nor variants of the gene.

In our study, we identified the loss of function (LOF) variant *NPRL3*: c.1137 dupT in I1, II3, II5, III2, which led to frameshift variant of proline at position 380. Premature termination codons (PTCs) appeared after 74 amino acids, which formed truncated proteins. The variant was at a non-polymorphic site and had not been documented in the gnomAD, ExAC, ESP6500, or 1,000 Genomes databases. The variant was reported to be related to FFEVF in the OMIM database (OMIM: 600928). Human Splicing Finder analysis showed that the *NPRL3* variant would cause difficulty in accurately identifying exons and affect the normal recognition and splicing of adjacent splice donor sites. Mutation Taster analysis predicted that *NPRL3*: c.1137 dupT was a “pathogenic vatiant”. Therefore, according to the American College of Medical Genetics and Genomics guidelines, the pathogenicity of the variant *NPRL3*: c.1137 dupT was “pathogenic” (PVS1+PM2+PP3).

### Differential RNA expression analysis of *NPRL3* variant

**Fig 2:**
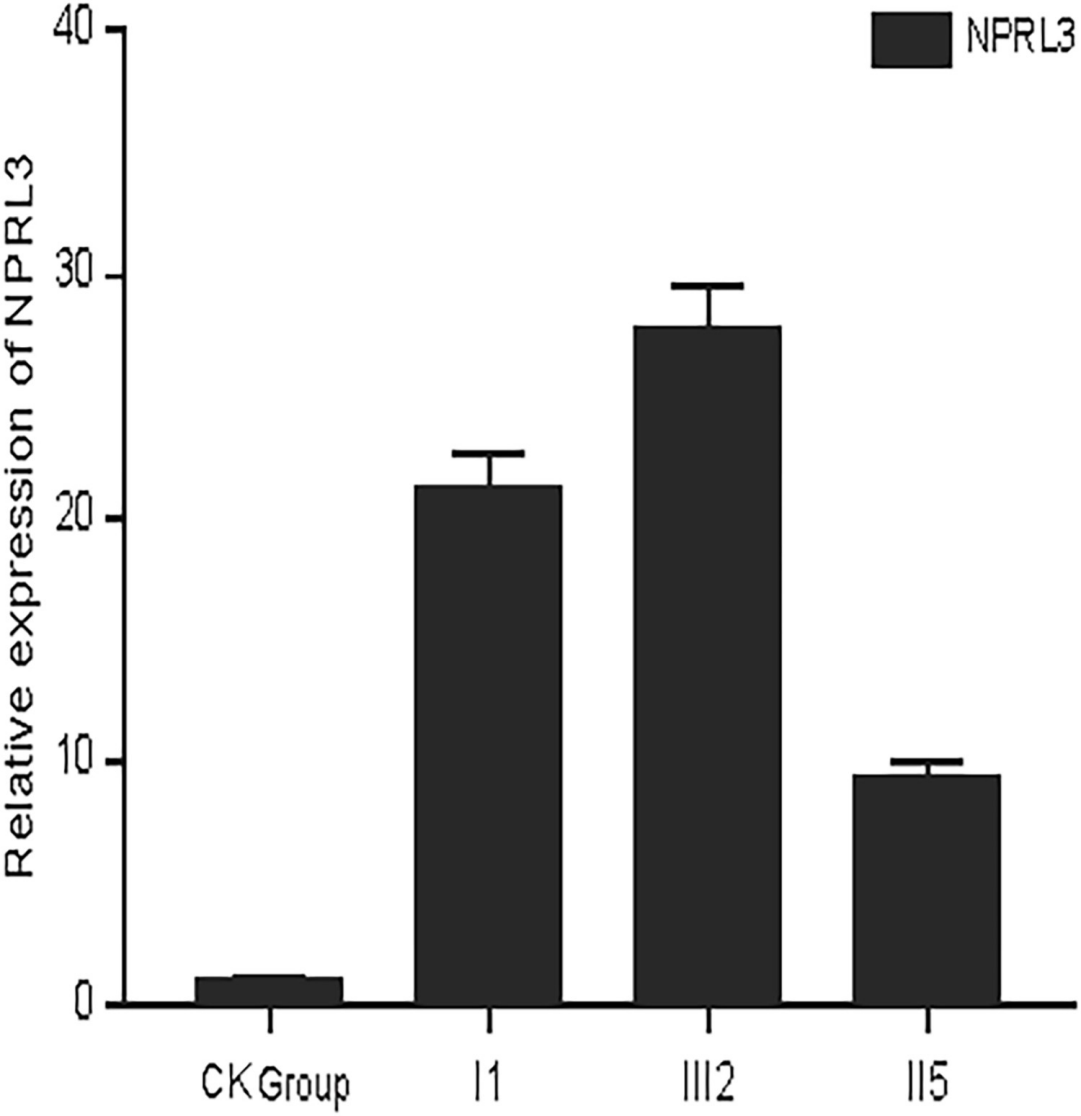
Relative expression level of *NPRl3*. Compared with the CK Group, the expression levels of I1, III2, and II5 in the study group were 21, 27, and 9 times, respectively.

The *NPRL3* expression levels in the study and control groups were calculated, and we found a much lower expression level in the control group than in the study group. The mRNA expression levels of the three individuals in the control group were averaged to constitute the Control Check Group (CK Group). We calculated the average Ct value (i.e., the number of cycles experienced by the fluorescence signal in each reaction tube when it reached the set domain value) and standard error of the mean value of the samples in the study and the CK Group (Table 2). Compared with the CK Group, the expression levels of I1, III2, and II5 were 21, 27, and 9 times, respectively (Fig 2).

**Table 2:** Amplification data analysis.

Target	Sample	Control	Expression	Expression SEM	Corrected Expression SEM	Mean Cq	Cq SEM
Actin	CK Group	*				20.68	0.04144
Actin	I1					26.93	0.09116
Actin	III2					26.78	0.06653
Actin	II5					26.87	0.01588
*NPRL3-1*	CK Group	*	1.00000	0.03659	0.03659	25.53	0.03270
*NPRL3-1*	I1		21.24440	1.44323	1.44323	27.37	0.03599
*NPRL3-1*	III2		27.94756	1.70825	1.70825	26.83	0.05788
*NPRL3-1*	II5		9.42736	0.51716	0.51716	28.49	0.07753

Abbreviations: SEM: Standard error of the mean; CK Group: Control Check Group.

### Verification of *NPRL3* transcription level by splicing in vivo

**Fig 3:**
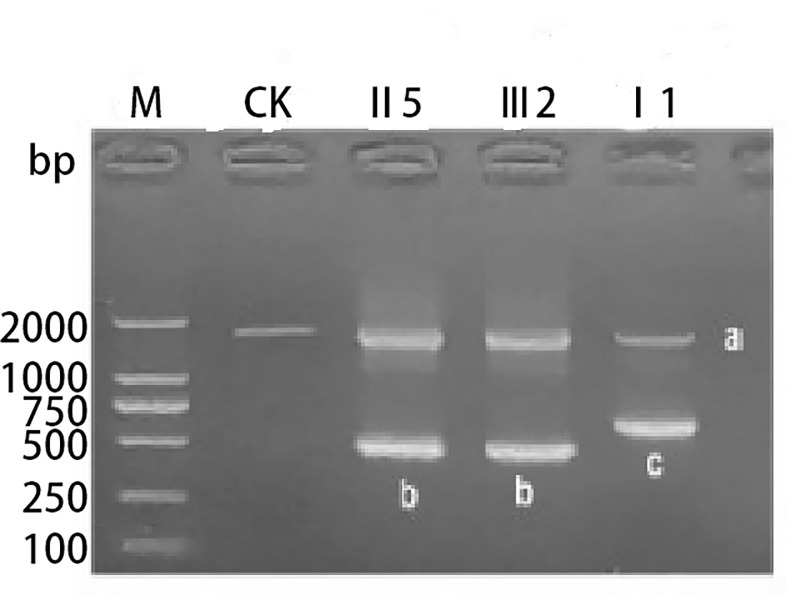
*NPRL3* reverse transcription-PCR results of the second round of amplification. The band of the expected size could be observed in the CK, while multiple bands in II5, III2, and I1.

**Fig 4:**
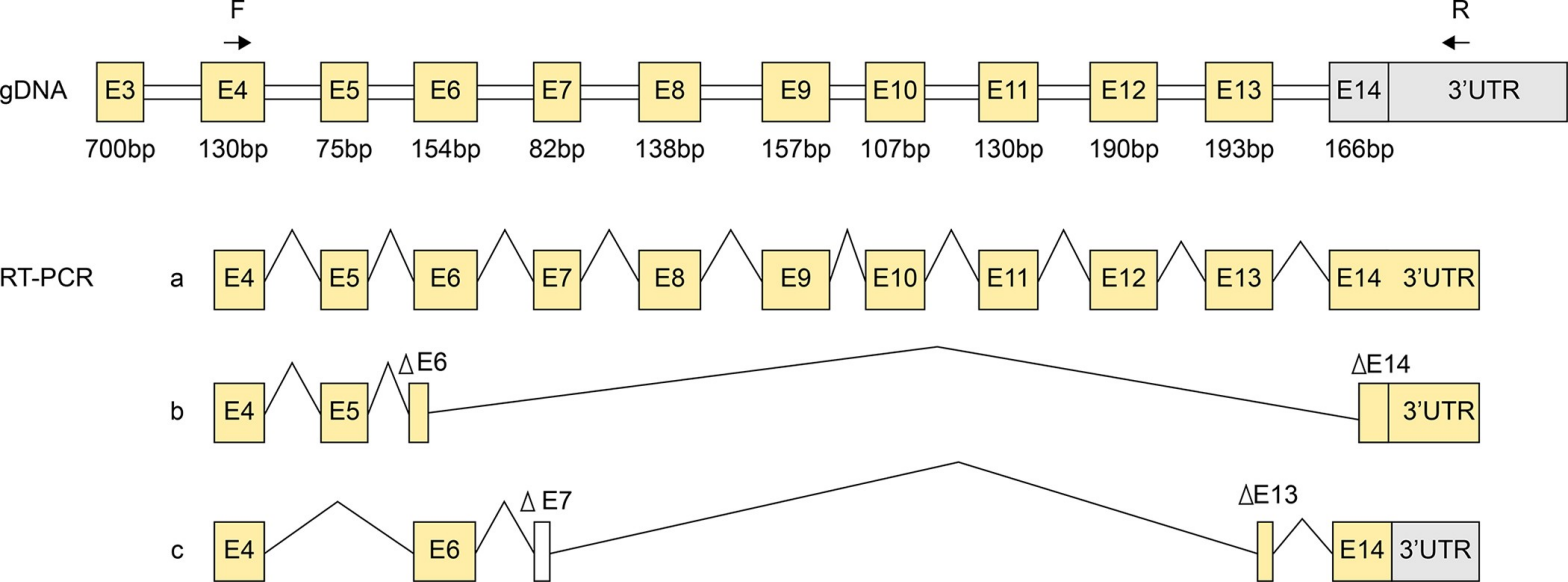
Splicing diagram of mRNA. The CK indicated normal splicing (band “a”). II5 and III2 had a normal splicing band “a” and another band “b”, which showed a missing large transcript segment, starting from 101 bp on the right side of Exon6 to 64 bp on the left side of Exon14. The splicing of I1 had a normal band “a” and another band “c” in which Exon5 jumps occurred in the transcript and large fragments were missing, starting from 64 bp on the right side of Exon7 to 149 bp on the left side of Exon13.

**Fig 5:**
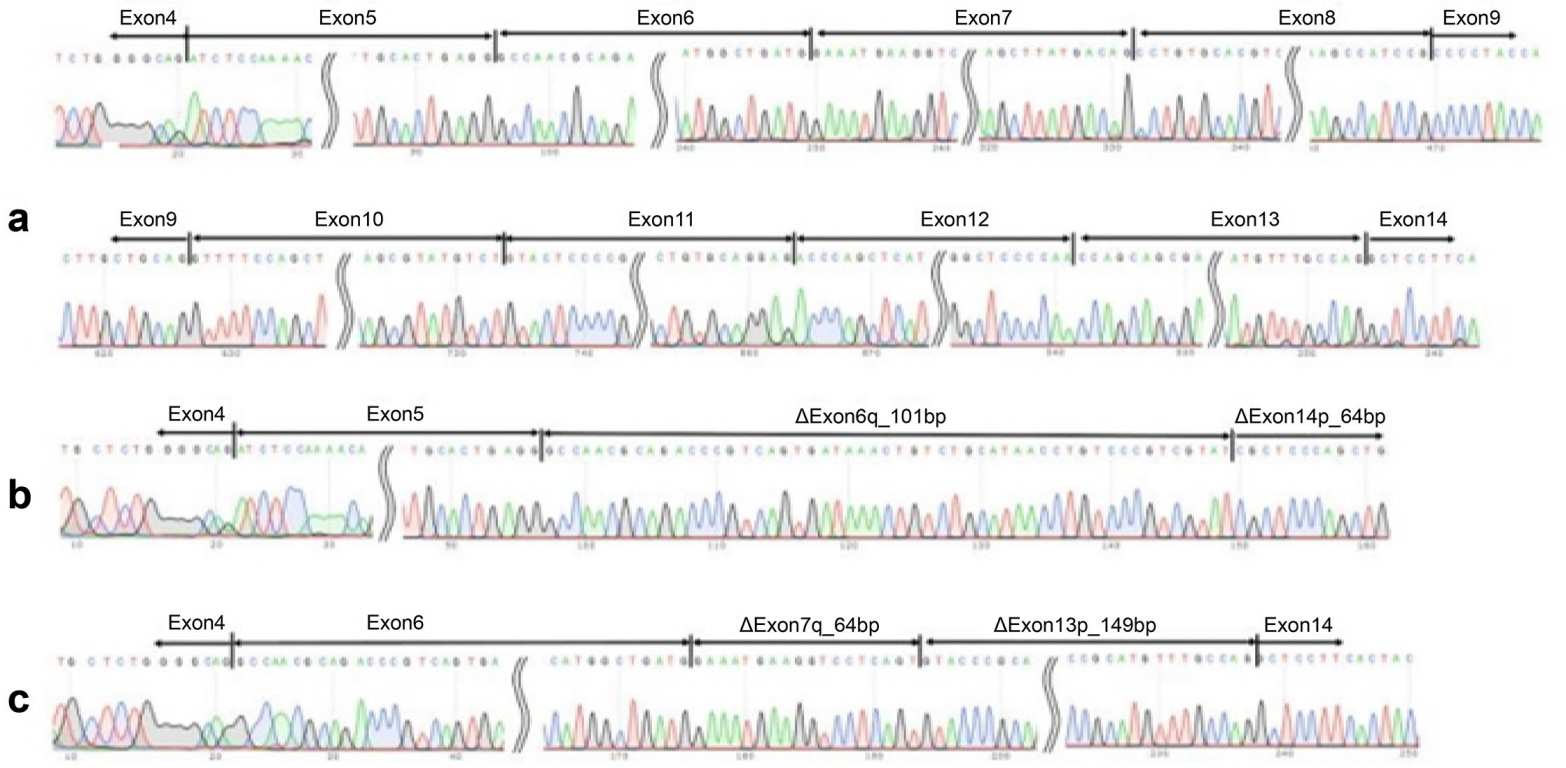
Splice sequencing results of mRNA.

After two consecutive rounds of nested reverse transcription-PCR amplification, the gel map showed the results of the second round of amplification (2F/2R). The band of the expected size (1655 bp) could be observed in the CK, while multiple bands were observed for II5, III2, and I1 (Fig 3). These bands were sequenced, and three splicing conditions were observed in the carriers (Figs 4 and 5).

The CK indicated normal splicing (band “a”): Exon4 (53 bp)–Exon5 (75 bp)–Exon6 (154 bp)–Exon7 (82 bp)–Exon8 (138 bp)–Exon9 (157 bp)–Exon10 (107 bp)–Exon11 (130 bp)–Exon12 (190 bp)–Exon13 (193 bp)–Exon14 (166 bp). II5 (heterozygous carrier) also had band “a” and another band “b”. Sequencing showed that a large transcript segment was missing in band “b”, starting from 101 bp on the right side of Exon6 to 64 bp on the left side of Exon14. The splicing mode of band “b” was as follows: Exon4 (53 bp)–Exon5 (75 bp)–△Exon6 (53 bp)–△Exon14 (102 bp). The splicing of patient III2 (heterozygous carrier) was the same as that of II5. The splicing of I1 (heterozygous carrier) also had band “a” and another band “c” in which Exon5 jumps occurred in the transcript and large fragments were missing, starting from 64 bp on the right side of Exon7 to 149 bp on the left side of Exon13. The splicing mode of band “c” was as follows: Exon4 (53 bp)–Exon6 (154 bp)–△Exon7 (18 bp)–△Exon13 (44 bp)–Exon14 (166 bp).

## Discussion

### Clinical features of FFEVF and genetic testing

FFEVF is a relatively rare form of autosomal dominant epilepsy with incomplete penetrance. Its symptoms vary greatly among members of the same family who usually have various epileptic foci. In our study, there were four affected family members, including three generations. They had a wide range of onset ages (infancy to youth) and different seizure types (focal to bilateral tonic-clonic seizures/focal seizures), onset sites (frontal lobe/temporal lobe), seizure times (daytime/night), and seizure frequencies (occasional /frequent). All patients showed epileptic interictal EEG discharge and there was even a slowed background rhythm in a patient (III2) with severe seizures. Although II3 denied having seizures, his EEG showed bilateral scattered low-amplitude sharp waves. He was therefore classified as a subclinical patient. Based on the symptoms above, the family was clinically diagnosed with FFEVF.

The clinical features of patients with different *NPRL3* variants reported from published papers [6,9,10] and our study have been listed in Table 3 to deepen our understanding of the newly discovered gene variant. Most patients had adolescent-onset disease. The seizure type differed, with some patients having more than one type of seizure. Treatment efficacy varied from patient to patient; some patients’ seizures were well controlled, while others did not respond well to anti-seizure medications. Epileptiform discharges in the interictal phase were found in some patients’ EEGs. However, our patients’ EEGs findings were significantly abnormal. In particular, I1 and III2 had EEG findings of paroxysmal θ waves, suggesting a certain degree of brain function decline, which could be related to recurrent seizures. The EEG findings of the patient with subclinical epilepsy (II3) were also abnormal, which suggested that for asymptomatic members with FFEVF, regular EEG follow-up might be helpful in early detection. The MRI findings varied; they could be normal or abnormal. The genetic testing findings are as follows: Weckhuysen et al. 2016 [6] found five patients had *NPRL3*: c.1270C>T, p.Arg424* and four patients had *NPRL3*: c.1070delC, p.Pro357Hisfs*56; Li et al. 2021 [9] found five patients had *NPRL3*: c316C>T, p. Q106*; Hu et al. 2022 [10] found two patients had *NPRL3*: c.954C>A, p.Y318* and one patient had *NPRL3*: c.1545-1G>C.

**Table 3:** Clinical features of patients with different *NPRL3* variants.

Ref	Age at seizure onset	Epilepsy type	Multiple seizure types	Seizure outcome	EEG	MRI
Weckhuysen et al.2016 [6]	7.08 yr (2 mo–26 yr)	2 GS, 1 Febrile convulsion, 3 FLE	1	1 seizure free, 4 ongoing seizures	1 N, 4 epileptiform discharges	2 N, 1 FCD+HS, 2 NA
Weckhuysen et al.2016 [6]	15.25 yr (2 yr–51 yr)	1 GS, 2 FLE, 1 TLE	0	3 seizure free, 1 ongoing seizure	1 N, 2 epileptiform discharges, 1 NA	1 N, 1 FCD, 2 NA
Li et al. 2021[9]	14.4 yr (8 yr-20 yr)	5 FBTCS	0	2 seizure free, 3 ongoing seizures	3 N, 2 epileptiform discharges	4 N, 1 brain atrophy
Hu et al. 2022 [10]	3.25 yr (1.5 yr-6 yr)	2 FBTCS	0	2 seizure free,	2 N	2 N
Hu et al. 2022 [10]	2 yr	2 FS, 1 FBTCS	1	1 ongoing seizure	1 epileptiform discharge	1 cortical thickening of the left frontal gyrus
Our study	15.8 yr (4 mo–31 yr)	3 FBTCS 3 FS	3	3 ongoing seizures, 1 no seizure	4 epileptiform discharges 1 slow background rhythm	4 N

Abbreviations: Ref: Reference; EEG: Electroencephalogram; MRI: Magnetic resonance imaging; yr: Years; mo: Months; GS: Generalized seizure; FLE: Frontal lobe epilepsy; TLE: Temporal lobe epilepsy; FBTCS: Focal to bilateral tonic-clonic seizure; FS: Focal seizure; N: Normal; NA: Not available; FCD: Focal cortical dysplasia; HS: Hippocampal sclerosis.

Because of the differences mentioned above, the severity of FFEVF varied greatly. Patients were likely to be misdiagnosed with sporadic epilepsy or other familial focal epilepsy forms until new affected members were identified, especially in smaller families. As the pedigrees were too small to demonstrate clear autosomal dominant inheritance, the prevalence of FFEVF was underestimated. In China, related research is rare, and there might be patients who did not have access to or could not undergo genetic testing. Therefore, it is necessary to deepen the understanding of FFEVF and carry out WES in suspected patients, especially those with a family history of focal epilepsy, as early as possible.

### Differential mRNA expression analysis of *NPRL3* variant

Currently, 140 GATORl complex variants have been reported in the literature. Among them, LOF variants, including nonsense variants and frameshift variants, account for 67%. LOF is considered to be one of the high-risk factosrs for focal epilepsy [11].

The *NPRL3* variant c.1137dupT is located in its INT domain, and the insertion variant will lead to a frameshift, resulting in the generation of PTC. There is a possibility of nonsense-mediated mRNA decay (NMD), which is a common post-transcriptional regulatory mechanism in many eukaryotes. As a monitoring system for mRNA, it can recognize mRNA with PTC and initiate degradation under the combined action of NMD factors [12,13],which prevent the production of pathogenic truncated proteins [14,15]. Dysfunction of NMD may be the molecular mechanism responsible for many neurological disorders such as epilepsy.

Our study found that the increased mRNA relative expression level was associated with the *NPRL3* variant. The mRNA relative expression level of the study group was significantly higher than the CK Group. We speculated whether there was a possibility of an NMD escape mechanism. Approximately 5%–25% of PTC-mRNA are known to escape NMD, which produces deleterious truncated proteins that ultimately lead to more severe clinical phenotypes [6]. In addition, compared with II5, who had mild symptoms, for I1 and III2, mRNA differences were more obvious than those in the CK Group. Whether this was meaningful should be further explored by functional experiments.

### Verification of *NPRL3* transcription level by splicing in vivo

Any step in genetic regulation may influence the phenotype of inherited diseases. Currently, the research hotspot in the exon region is focused on the effect of gene variants on encoding proteins. However, due to the limitations of research methods, the genetic variants affecting splicing have not been taken seriously. According to a previous study, 15%–30% of pathogenic gene variants affect RNA splicing, sometimes more than 50% [16].

Splicing regulatory elements such as exonic splicing enhancers (ESE) and exonic splicing silencers (ESS) can promote the accurate recognition of exons in splicing [16]. When they are mutated, abnormal splicing may occur, such as exon loss, intron retention, activation of potential splicing sites. This results in structural and functional abnormalities of protein products or down-regulation of normal product expression, ultimately leading to the occurrence of diseases.

Human Splicing Finder analysis showed that the *NPRL3* variant in our study was in ESE, which would destroy the original ESE and generate new ESS. It resulted in difficulty in accurately identifying exons and then affected the normal recognition and splicing of adjacent splice donor sites.

As shown in Figs 4 and 5, all three patients had a similar splicing “a” band. Abnormal splicing “b” band was detected in II5 and III2: Exon4 (53 bp)–Exon5 (75 bp)–△Exon6 (53 bp)–△Exon14 (102 bp). However, the “c” band splicing mode of patient I1 was different from those of II5 and III2: Exon4 (53 bp)–Exon6 (154 bp)–△Exon7 (18 bp)–△Exon13 (44 bp)–Exon14 (166 bp).

Compared with the symptoms of II5 and III2, those of I1 were characterized by a late onset age, frequent nocturnal seizures, and anti-seizure medications refractoriness, This suggested that splicing regulation might be an important factor affecting phenotypes.

Hu et al. [10] identified another splicing variant, *NPRL3*: c.1545-1G>C, in a Chinese family with FFEVF. FFEVF might be associated with abnormal splicing, resulting in the loss of the last exon of *NPRL3*. Iffland et al. [17] evaluated the brain tissue of patients with *NPRL3* c1375_1376dupAC and reported findings of focal cortical dysplasia and increased cell size. They indicated that *NPRL3* knockdown led to alterations in the cell morphology of mouse neuronal cell lines in a mTOR-dependent manner and altered the subcellular localization of mTOR in neural precursor cells in vitro, further suggesting the critical role of *NPRL3* in neurons.

At present, few Chinese studies on *NPRL3* variants in families with FFEVF have used multiple molecular analysis methods. Li et al.[9] confirmed a novel pathogenic variant in *NPRL3*: c316C>T; p. Q106*. After the pathogenicity of the gene variant was predicted via in silico analysis, in vitro experiments (PCR, Western blotting, and immunohistochemistry) were conducted to analyze the gene transcription, protein expression, and subcellular localization of *NPRL3* and related signaling molecules in the peripheral blood cells of the family members. Hu et al. [10] identified *NPRL3*: c.954C >A, p.Y318* and the splicing variant *NPRL3*: c.1545-1G>C. After the pathogenicity of the gene variants was further assessed in different algorithms, the researchers used the Self-Optimized Prediction Method from Alignment (SOPMA) to predict the effect of the identified variant on the secondary structure of NPRL3 and SWISS-MODEL software to investigate the effect of the variant on the 3D structure of NPRL3.

Our study has expanded the phenotype spectrum of FFEVF and found a new *NPRL3* variant that had not been reported. Compared with unrelated healthy controls, we found that the *NPRL3* variant was associated with the relative expression level of mRNA and abnormal splicing in vitro. However, the case-control design of the study involving unrelated healthy controls is highly underpowered for evaluating the pathogenicity of the variant. Moreover, other members of the family refused to provide blood samples due to privacy concerns. Therefore, only one unaffected family member was included in the genetic analysis. Consequently, it was difficult to calculate the penetrance accurately. A detailed analysis of the relationship between gene variants and clinical phenotypes has not been conducted yet.

In the future, we shall continue to follow up with each patient. More unaffected family members will be recruited, which could enable assessment of the relationship of the variants with focal epilepsy. Relevant functional studies, such as minigene report detection, will be conducted to provide clues for the in-depth study of FFEVF, which is important for the accurate diagnosis and treatment of FFEVF.

## Supporting information

S1 Raw images (TIF)
