# Supplementary figures and images for: The clinical features of familial focal epilepsy with variable foci and NPRL3 gene variant
Source: PLoS One. 2023 Apr 26;18(4):e0284924. doi: 10.1371/journal.pone.0284924 (PMC10132533; doi:10.1371/journal.pone.0284924)

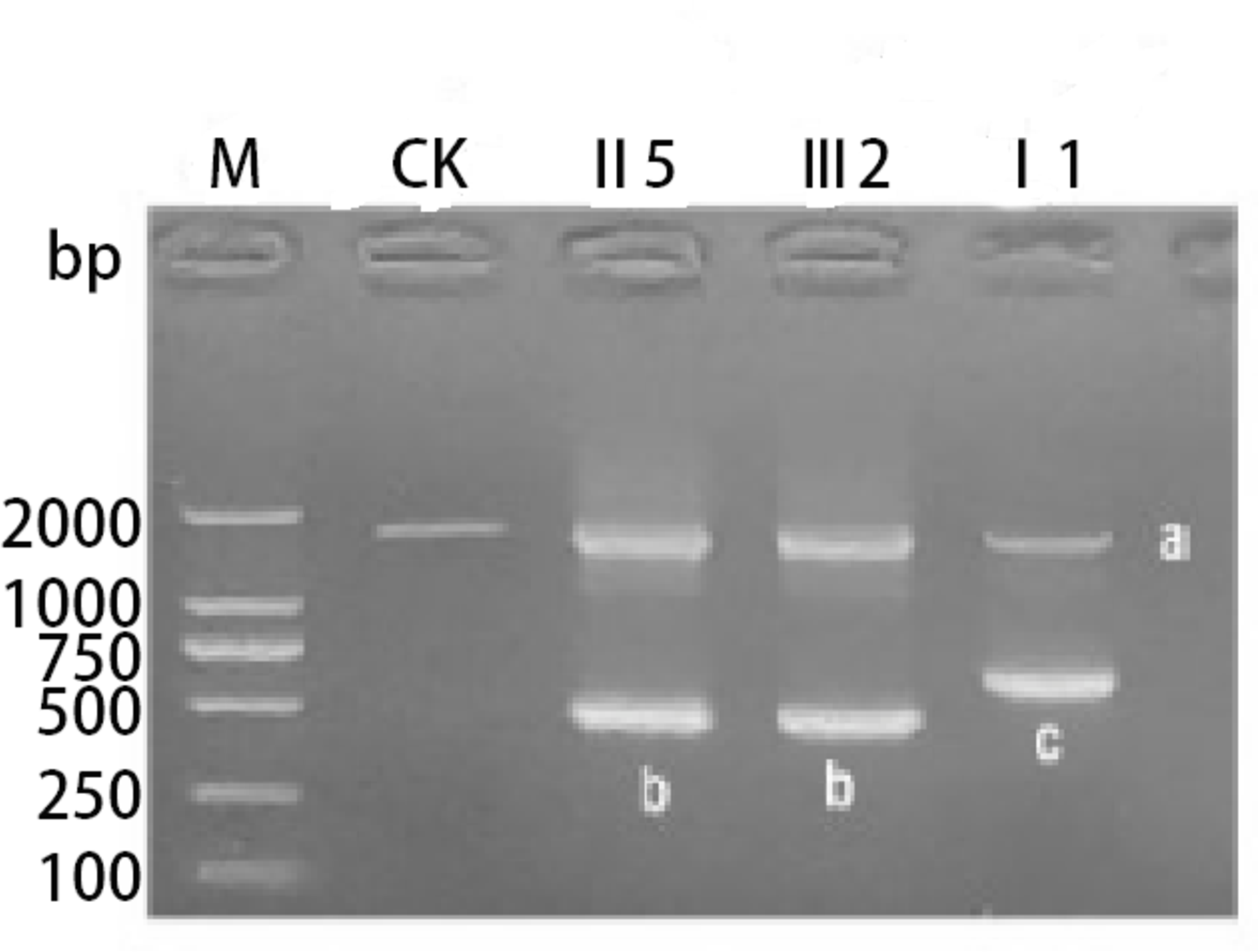

Supplement: S1 Raw images — (TIF) [file pone.0284924.s001.tif]
